# Chlorhexidine body washing to control antimicrobial-resistant bacteria in intensive care units: a systematic review

**DOI:** 10.1007/s00134-012-2542-z

**Published:** 2012-04-12

**Authors:** Lennie P. G. Derde, Mirjam J. D. Dautzenberg, Marc J. M. Bonten

**Affiliations:** 1 Julius Center for Health Sciences and Primary Care, University Medical Center Utrecht, Universiteitsweg 100, 3584 CG Utrecht, The Netherlands; 2 P.O. Box 85500, 3508 GA Utrecht, The Netherlands

**Keywords:** ICU, Chlorhexidine body washing, Systematic review, AMRB

## Abstract

**Purpose:**

Infections caused by antimicrobial-resistant bacteria (AMRB) are increasing worldwide, especially in intensive care units (ICUs). Chlorhexidine body washing (CHG-BW) has been proposed as a measure to limit the spread of AMRB. We have systematically assessed the evidence on the effectiveness of CHG-BW in reducing colonization and infection with AMRB in adult ICU patients.

**Methods:**

PubMed, Embase, CINAHL, and OpenSigle databases were searched using synonyms for “intensive care unit,” “hospital,” and “chlorhexidine.” All potentially relevant articles were examined by two independent reviewers. Inclusion was limited to studies with ICU patients as domain, providing outcomes related to colonization or infection with AMRB. Data from 16 studies were extracted; 9 were excluded because of assessed high risk of bias or inadequate analyses. The remaining studies differed markedly in (co-)interventions and case mix, which precluded pooling of data in a formal meta-analysis.

**Results:**

Incidences of MRSA acquisition were reduced significantly in three studies in which this was the primary endpoint. Significant reduction in MRSA infection rates was observed in only one of five studies. Carriage and bacteremia rates of VRE were assessed in one study, and both significantly declined. There were hardly any data on the effects of CHG-BW on antibiotic-resistant gram-negative bacteria (ARGNB).

**Conclusions:**

CHG-BW may be effective in preventing carriage, and possibly bloodstream infections, with MRSA and VRE in different ICU settings. As CHG-BW protocols, co-interventions and case mix varied widely, attribution of these effects to CHG-BW alone should be done with care. Evidence that CHG-BW reduces carriage of or infections with ARGNB is lacking.

## Introduction

The failing control of antimicrobial-resistant bacteria (AMRB) is an important and continuously growing threat to the delivery of adequate medical care in hospitals and the community [1]. Infections caused by AMRB usually require longer and more complex treatments than those caused by susceptible bacteria [2, 3]. Nosocomial infections with AMRB are associated with delayed initiation of appropriate therapy, failure of therapy, prolonged length of hospital stay, and increased mortality.

Patients admitted to the intensive care (ICU) are extremely prone to infections, including those caused by AMRB. Main contributing factors are underlying immunodeficiency, co-morbidities, use of invasive devices, and the intensity of patient care. These factors, combined with extensive use of antibiotics, facilitate patient-to-patient transfer of AMRB [4].

Chlorhexidine gluconate (CHG) is a cationic bis-biguanide developed in the UK around 1950. Recently, there has been a renewed interest in this antiseptic as a measure to prevent infections with, and transmission of, AMRB in ICU patients. Cross-transmission of AMRB is extremely important in the dynamics of these bacteria, and temporarily contaminated hands of health care workers are considered the most important vectors for spread [5]. AMRB frequently colonize the skin of ICU patients, and decontamination of these body surfaces may not only prevent development of infections but also reduce the potential for cross-transmission.

We aimed to evaluate the evidence for the effectiveness of chlorhexidine body washings (CHG-BW) in reducing colonization and infection with AMRB in adult ICU patients, measured as colonization or infection with methicillin-resistant *Staphylococcus aureus* (MRSA), vancomycin-resistant *Enterococci* (VRE), and/or antibiotic-resistant gram-negative bacteria (ARGNB). We assessed the effect, when possible, on different AMRB separately, as the effect might differ between bacterial species. We focused on CHG-BW, and not on the use of CHG for oral decontamination and pre-surgical skin preparation, which have been systematically reviewed recently [6, 7].

## Materials and methods

### Search strategy

Methods and inclusion criteria of the review were specified in advance and documented in a protocol (see “Appendix”). All studies in PubMed, Embase, CINAHL, and OpenSigle from their inception to 1 April 2011 were considered. Databases were searched using “intensive care unit” and “hospital” (all variants and abbreviations) with Boolean “OR” to describe the setting and “chlorhexidine” OR “body wash” to describe the intervention. In OpenSigle, only “chlorhexidine” was used as search term to include all possibly relevant studies. We included all studies of adult ICU patients that investigated CHG-BW as an intervention to control AMRB and had colonization, clearance of colonization, or infection as an outcome. Non-English language papers were accepted if they fulfilled the above-mentioned criteria. A related article and reference search was performed.

### Data collection and selection of studies

Duplicates were removed, and the title and abstract of all identified articles were screened for relevance, without blinding to journal and authors, by two independent reviewers (LD and MD). In case of discordant results consensus was reached by discussion with a third reviewer (MB). Reviews were included if there was any reason to assume that original data were present. Letters to scientific journals were not automatically excluded, as they could contain original data. Outbreak reports (an outbreak was defined as an increase in incidence lasting <6 months) were excluded, as success in outbreak situations cannot be generalized to non-outbreak situations. Chart reviews were also excluded. Studies were eligible if the setting was the ICU, or hospital without explicit absence of an ICU, and if outcomes were related to colonization or infection with AMRB.

All potentially relevant articles were obtained, and the full text was examined. Because of the high proportion of studies with low quality design, a high possibility for bias or the absence of a control group, we decided at this point to limit inclusion to randomized controlled trials (RCTs) and interrupted time series (ITS) design with three or more time-points. In before–after design studies, proper ITS analyses and at least three time points before and after initiation of the intervention are recommended [8, 9]. Even then, there is a possibility that the internal validity of ITS is compromised by trends that are already present before the start of intervention, outcomes that are measured differently over time, and differential dropout in the intervention group. Therefore, we only selected ITS adhering to the recommendations of the Cochrane “Effective Practice and Organization of Care” Group (EPOC) [8] in order to limit these threats to internal validity.

**Fig. 1:**
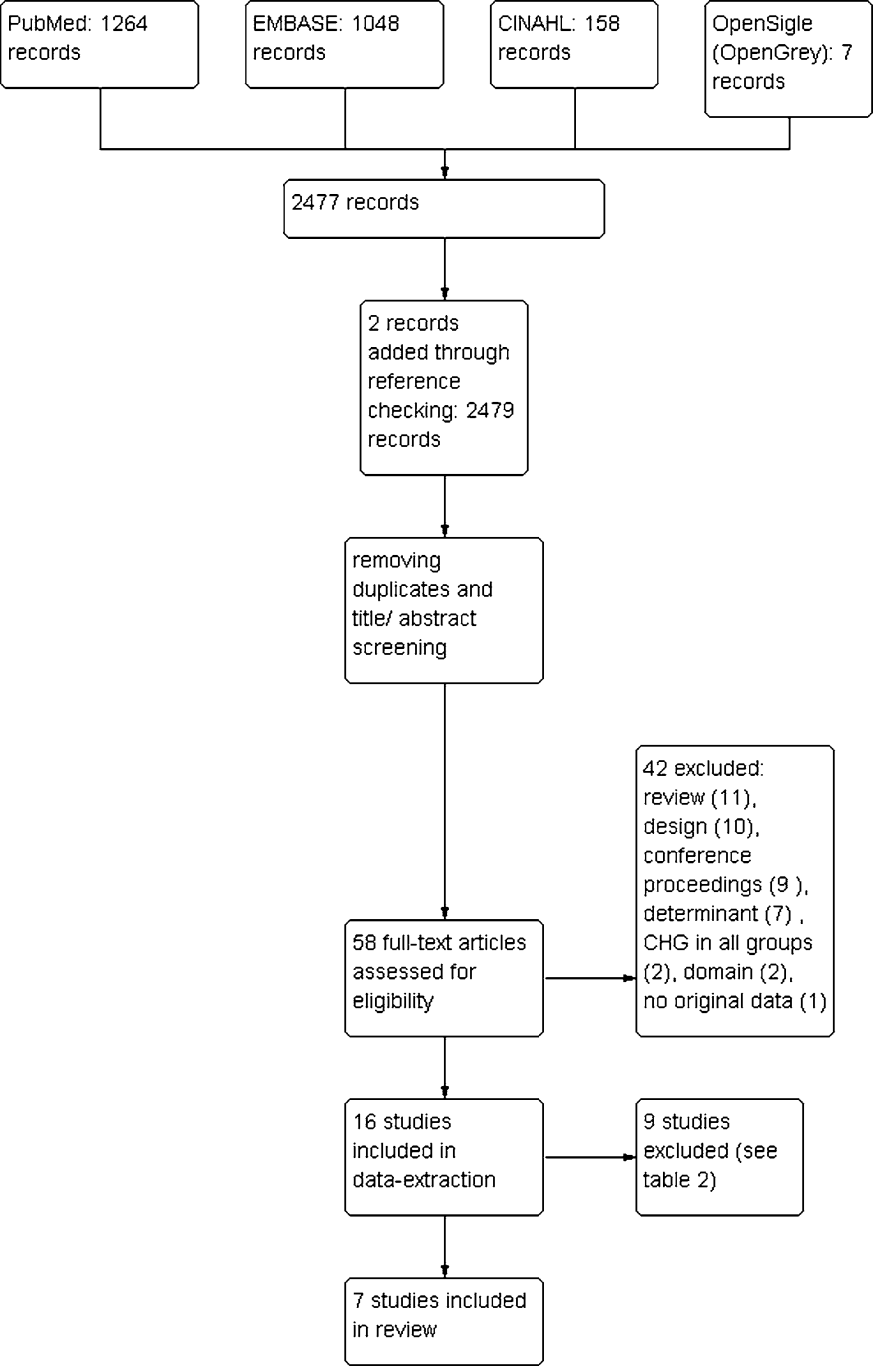
Study flow diagram

None of the studies including hospital patients included results for ICU patients separately, nor could these results be calculated from the data presented. Therefore, we excluded these studies. The data collection flowchart is shown in Fig. 1.

### Data extraction and management

For each of the 16 included studies, the following characteristics were extracted: design, setting, domain, co-interventions, outcome(s), possible sources of bias, missing data, and the statistical analyses used to evaluate the outcome(s). Only effect measures related to colonization and/or infection were included.

Only one study (one out of two RCTs) used the ICU rather than the individual patient as unit of analysis [10]. Because of this sparseness of RCTs using unit-based analyses, we did not exclude any studies based on this issue. As many studies did not mention missing data, nor the way missing data were handled, this was only assessed when present. Because of heterogeneity in designs, no meta-analyses to obtain pooled results could be performed.

## Results

Our search yielded 2,477 abstracts; two extra records were retrieved by related article and reference search [11, 12]. In both articles, neither “chlorhexidine” nor any of the other search terms were mentioned in the title or abstract. Seven studies were included in the final review (Table 1) [10, 13–18]. One of these studies investigated two interventions in a 2 × 2 factorial design. Patients (*n* = 515) were randomized to receive topical antibiotics (polymyxin and tobramycin; applied in the oropharynx and through the nasogastric tube) or placebo, and also to receive mupirocin ointment in the nose and CHG-BW or placebos. For the current review we only used the data from the patient groups that did not receive topical antibiotics, but CHG-BW with mupirocin (*n* = 130) or placebos (*n* = 126). Of nine excluded studies, seven were excluded based on inadequate analyses of ITS studies (following EPOC criteria) [19–25]. In one RCT and one ITS, insufficient data were present to calculate the effectiveness of CHG-BW (Table 2) [26, 27].

**Table 1:** Characteristics of included studies

Study	Design	Patients included (*n*)	Duration (months)	Setting	Domain^a^	CHG intervention	Co-interventions or control group	Primary outcome	Secondary outcome(s)
Batra [13]	ITS	4,570	51	Single center, mixed ICU	Patients colonized or infected with MRSA	1 % CHG in nostrils/mouth/tracheotomy site QID	Educational campaign (reinforcing HH and barrier nursing, covert HH and barrier nursing audit and monthly MRSA infection rate feedback)	Transmission of MRSA colonization	–
						1 % CHA in groin/axillae/skin folds daily	MRSA colonized nursed in side rooms or pairs		
						4 % CHG body wash daily			
Bleasdale [10]	RCT	836	12	Single center, medical ICU	All patients	2 % CHG body wash daily with impregnated cloths	Daily bathing with soap and water	All-cause primary BSIs	All-cause UTI, VAP, and secondary BSIs.
Camus [14]	RCT	256^b^	30	Multi-center, medical ICUs	Patients with expected duration of ventilation > 48 h	15 ml of 4 % CHG body wash every 12 h for 5 days with or without SDD	SDD plus “body wash placebo” or placebo only	All-cause infections acquired until 48 h after termination of study treatments	All-cause total and device-related infections
Climo [15]	ITS	5,043	12	Multi-center, medical, surgical, cardiac surgery; and coronary/medical ICUs	All patients	4 % CHG body wash daily	Daily bathing with non-medicated soap and water	Acquisition of MRSA and VRE colonization and BSIs	–
Gould [16]	ITS	2,653	48	Single center, mixed ICU	All patients	4 % CHG body and hair wash daily	Nasal ointment QID (a) 2 % fusidic acid; (b) 3 % oxytetracycline (only available first 6 months) or (c) 0.5 % neomycin sulphate w 0.1 % chlorhexidine hydrochloride	Acquisition of MRSA colonization and infection	*S. aureus* bacteremia
Popovich [17]	ITS	3,048^c^	24	Single center, surgical ICU	All patients	2 % CHG body wash daily	Daily bathing with bar soap, warm water and cotton washcloths	Acquisition of all-cause CLABSIs	Acquisition of other nosocomial infections
Raineri [18]	ITS	3,978	120	Single center, mixed ICU	All patients	4 % CHG body wash daily for 5 days	Post-intervention education session for new HCWs, monthly infection control meetings, strict isolation, and cohorting	Acquisition of MRSA colonization and infection	–
						CHG shampoo on day 1 and 5			

*BSI* bloodstream infection, *CHA* chlorhexidine acetate, *CHG* chlorhexidine gluconate, *CLABSI* catheter-related bloodstream infections, *ICU* intensive care unit, *ITS* interrupted time series, *MRSA* methicillin-resistant *Staphylococcus aureus*, *RCT* randomized controlled trial,* S. aureus* *Staphylococcus aureus*, *SDD* selective digestive decontamination, *UTI* urinary tract infection, *VAP* ventilator-associated pneumonia, *VRE* vancomycin-resistant *Enterococci*

^a^The domain for the CHG intervention is stated. The domain for co-interventions can differ

^b^Only taking into account the “neither” and the “CHG” regimen patients

^c^Calculated from mean monthly admission rate of 138 during 12 months at baseline and 116 during 12 months at intervention phase

**Table 2:** Characteristics of excluded studies

Study	Design	Reason for exclusion
Dixon [19]	ITS	Does not use time-series analysis. Source and method of data collection not mentioned
Dryden [26]	RCT	CHG-BW was used in both groups
Evans [20]	ITS	Does not use time-series analysis
Fraser [21]	ITS	Does not use time-series analysis
		ITS with only one data point per period
Holder [22]	ITS	No formal analysis, only descriptive data. High risk of bias (regression to the mean)
Munoz-Price [23]	ITS	Does not use time-series analysis. Possible regression to the mean. Risk of reporting bias (the “unblinded” preventionist reported the number of infections). Substantial non-compliance, not quantified in intervention period
Popovich [24]	ITS	Does not use time-series analysis
Ridenour [25]	ITS	Does not use time-series analysis
Robicsek [27]	ITS	Not suitable to assess effectiveness of CHG-BW (focus on different types of surveillance)

*CHG-BW* chlorhexidine gluconate body washing, *ITS* interrupted time series, *RCT* randomized controlled trial

### Quality and completeness of the evidence

Of seven included studies three determined acquisition rates of MRSA carriage [13, 15, 16]; one determined acquisition rates of VRE carriage [15]. Five quantified MRSA [14–18] and one quantified VRE infection rates [15]. Four studies reported (limited) results on infections with ARGNB [10, 14, 16, 17].

Co-interventions were used in four studies [13, 14, 16, 18], and CHG-BW protocols as well as patient case mix differed extensively between studies (Table 1).

Compliance with CHG-BW protocol was measured in one study only [15]. In this study, actual use of CHG was compared to predicted use, and coordinators urged better compliance if needed. However, compliance data were not presented. Hand hygiene compliance was not systematically assessed in any of the studies.

### Risk of bias in included studies

There was no perceived risk of selection bias in the selected studies. In one study, 3,928 of 4,444 admitted ICU patients were excluded, but only 4.6 % of these patients were excluded for other reasons than those stated in the exclusion criteria [mainly because of logistic issues on weekends (3.7 %)] [14].

A double-blind design was used in one [14] and partial blinding in another RCT in which one of three investigators categorizing bloodstream infections (BSIs) and the category adjudicator were blinded to the study arm [10]. Naturally, blinding was not used in the studies with ITS design.

Possibilities for detection bias were considered present in three studies [13, 15, 16]. In one study the method of screening changed during the trial [13]; in another one compliance with obtaining surveillance cultures increased during the study [15], and in the third study screening cultures were used during intervention, but not during the baseline period [16]. In the latter two studies, though, detection bias may have underestimated the effectiveness of the intervention, as detection of the primary endpoint improved after the intervention was implemented.

Attrition bias was not considered relevant in any of the included studies. In one study 3 of 391 patients in the CHG-BW arm did not receive bathing because of skin rashes (eventually considered as not related to CHG-BW), and these patients were included in the intention-to-treat analysis [10]. In another study 1 of 126 patients in the placebo group was withdrawn from the analysis because of premature unblinding [14].

Though no formal meta-analysis was performed, the presence of studies with negative results demonstrates that publication bias was not complete.

Selective outcome reporting may have been present, but was difficult to assess as study protocols for studies using an ITS design were not available. Protocols were available for the two RCTs. For one RCT the protocol, as accessed through http://www.clinicaltrials.gov, stated that microbiological data were collected, but only data related to BSIs were reported [10]. The authors stated that these data will be published separately. The protocol of the other RCT was kindly provided by the authors, and no risk of selective outcome reporting was detected [14]. For the ITS studies, we compared information in the “methods” sections to the “results” sections, and evidence of selective outcome reporting was not detected. In one of these studies, it was stated that the intervention was not part of a pre-planned study protocol [17].

### Effects of interventions

Incidences of acquisition of MRSA carriage were reduced significantly in the three studies in which this was the primary endpoint (Table 3) [13, 15, 16].

**Table 3:** Summary of findings

Study	Patients included (*n*)	Duration (months)	Infection	Colonization
Batra [13]	4,570	51		70 % reduction in acquisition of endemic MRSA strains (rate ratio 0.3), but increased acquisition (rate ratio 3.85) with an outbreak MRSA strain
Bleasdale [10]	836	12	61 % incidence reduction in all-cause primary BSIs; rate difference 6.3/1,000 ptdays 16.8 versus 6.4 BSIs per 1,000 central line-days (*p* = 0.01)	
			No significant reduction in all-cause UTI, VAP, and secondary BSIs	
Camus [14]	256	30	No significant reduction in all-cause ICU-acquired infections (*p* = 0.919)^a^	
			No significant reduction in all-cause total infections^a^	
			No significant reduction in all-cause device-related infections^b^	
Climo [15]	5,043	12	No reduction in MRSA bacteremia^c^	25 % reduction in acquisition of MRSA colonization (−0.66 per 1,000 ptdays)^c^
			78 % reduction in ICU acquired VRE bacteremias (−2.64 per 1,000 ptdays)^c^	45 % reduction in acquisition of VRE colonization (−1.51 per 1,000 ptdays)^c^
Gould [16]	2,653	48	No significant reduction in MRSA or MSSA bacteremia	11.4 decrease (*p* = 0.005) in proportion of patients with MRSA (colonization or infection)
Popovich [17]	3,048	24	No significant reduction in ICU-acquired all-cause CLABSIs (*p* = 0.57)	
			Significant decrease in incidence rate of MRSA clinical cultures (0.68 versus 1.03 per 1,000 ptdays, *p* = 0.49)	
			No significant reduction in ICU-acquired other infections (all *p* values >0.18)	
Raineri [18]	3,978	120	Decrease of MRSA infection rate from 3.5 to 1.7 per 1,000 ptdays (*p* = 0.0023)	
			No significant difference in MRSA-VAP	
			Decrease in MRSA-BSI incidence rate from 1.65 to 0.29 cases per 1,000 ptdays (*p* = 0.02)	

*BSI* bloodstream infection, *CHG* chlorhexidine gluconate, *CHG-BW* chlorhexidine gluconate body washing, *CLABSI* central line-associated bloodstream infection, *MRSA* methicillin-resistant *Staphylococcus aureus*, *PO* primary outcome, *Ptdays* patient-days, *SO* secondary outcome, *TW-MRSA* sequence type 239 MRSA outbreak strain, *UTI* urinary tract infection, *VAP* ventilator-associated pneumonia, *Vent days* ventilator days, *VRE* vancomycin-resistant *Enterococci*

^a^There was a significant effect for the polymyxin/tobramycin plus CHG/mupirocin group when compared to each regimen alone and neither regimen

^b^There was also no significant difference for the polymyxin/tobramycin plus CHG/mupirocin group when compared to each regimen alone and neither regimen

^c^Only the results of the time-series analysis are presented

^d^For period 1 compared to period 2. For the whole trial (period 1 to period 3), there was a significant decrease (*p* = 0.006 for trend)

MRSA infection rates were a primary outcome in three studies [15, 16, 18], and two studies presented limited data on MRSA infection rates [14, 17]. A statistically significant incidence reduction was observed in one [18]. Two studies failed to demonstrate statistically significant effects on MRSA bacteremia, although MRSA-carriage rates decreased in both studies [15, 16]. Absolute numbers of MRSA bacteremia, though, were only 40 (29 before and 11 after intervention) and 13 (8 before and 5 after intervention) in these studies. MRSA infections were even lower in the studies in which this was not a primary outcome. In one study there were two and five MRSA infections in the CHG-BW and placebo group [14], and in the other study there were five and six clinical cultures yielding MRSA at baseline and during the intervention, respectively (incidence rate of 0.68 vs. 1.03 per 1,000 patient-days; *p* = 0.49) [17].

Carriage and bacteremia rates due to VRE were analyzed in one study; these were reduced by 45 and 78 %, respectively [15].

Reported results of CHG-BW on preventing all-cause infections were more heterogeneous. There was a statistically significant 61 % decline in the incidence of all-cause primary BSIs in one study [10], whereas no significant reductions in central line-associated BSIs (CLABSIs) were reported in two other studies [14, 17].

Although incidences of colonization and/or infections with ARGNB were not primary outcomes in any of the studies, some results were provided. In one study, 1 out of 27 and 2 out of 11 primary BSIs were caused by gram-negative bacteria before and after the introduction of CHG-BW, respectively [10]. In another study, 5 out of 13 and 1 out of 12 clinical cultures grew imipenem-resistant *A. baumannii* before and during the use of CHG-BW, respectively, although overall more CLABSIs were due to gram-negative bacteria (and yeasts) during CHG-BW [17]. In a third study, the number of patients acquiring infections with gram-negative bacteria was 50 of 126 randomized to placebo and 44 of 130 randomized to CHG-BW plus nasal mupirocin, without further information on antibiotic susceptibilities [14]. In a fourth study, carriage and bacteremia rates with ARGNB were 1 % or lower in both study periods [16]. Therefore, there was hardly any evidence on the effects of CHG-BW on carriage with ARGNB.

## Discussion

The results of this systematic review demonstrate that CHG-BW may be effective in preventing bloodstream infections and carriage with MRSA and VRE in different ICU settings. This conclusion is based on seven studies with good methodological quality and low risk of bias, but marked differences in interventions, co-interventions and patient case mix, which precluded pooling of data in a formal meta-analysis.

Though much can be learned from less robust studies, like outbreaks, we chose methodological selection criteria to select only the best available evidence. Before-after studies not fulfilling these criteria have a high chance of inappropriately attributing the found effect to the intervention, as they do not correct for baseline trends [8].

In four studies, co-interventions were present, such as the use of mupirocin intranasally, active surveillance cultures, isolation or other barrier precautions, and education programs [13, 14, 16, 18]. Therefore, attribution of the beneficial effects on infections and carriage with MRSA and VRE to CHG-BW alone should be made with care.

There was no evidence (nor lack of evidence) that CHG-BW reduces acquisition of carriage or infections with ARGNB. CHG works by attachment to and disruption of cytoplasmic membranes of bacteria, and should, therefore, be effective against gram-positive and -negative bacteria [5]. In vitro, though, CHG has slightly better activity against gram-positive bacteria [5].

Possible adverse events and the emergence of resistance against CHG are important issues that were not systematically assessed. However, no severe allergic skin reactions were reported in the included studies. In one study slightly higher median minimally inhibitory concentrations to CHG were observed among blood culture isolates during CHG-BW, compared to soap-and-water bathing, but this difference was attributed to isolation of fewer (very susceptible) gram-positive bacteria during CHG-BW rather than to an increase in the absolute number of bacteria with elevated minimally inhibitory concentrations for CHG [10].

Since decontamination of body surfaces may not only prevent development of infections but also reduce the potential for cross-transmission, CHG-BW may influence the risk of non-treated patients to acquire bacterial carriage (i.e., colonization pressure) [28]. The effects of CHG-BW are, therefore, best evaluated when applied to all patients in a unit simultaneously, and individual patient randomization may not be the most appropriate study design. There was only one RCT in which the effectiveness of CHG-BW was evaluated on the unit level [10].

The most practical approach for unit-based interventions is a before–after study. Unfortunately, from a methodological perspective, this is a weak study design because of intrinsic risks of bias [9]. Moreover, not incorporating patient dependency in the statistical analysis may lead to wrong inferences [29]. Therefore, seven studies employing ITS design, but not complying with EPOC guidelines were excluded from our analyses [19–27].

In a recent meta-analysis of 12 studies investigating the effects of CHG-BW on the incidence of BSIs, no methodological criteria were applied for study selection [30]. Five of those studies were also included in our study, [10, 14–17], and reductions in BSIs were apparent in three [10, 15, 16]. However, our study adds important nuances to the conclusions of O’Horo et al. In one of the abovementioned three studies, only a reduction in primary, but not in secondary all-cause BSIs was apparent [10]; in one study a significant reduction in ICU-acquired VRE bacteremia, but not of MRSA bacteremia was demonstrated [15], and in the remaining study there were no reductions in MRSA and MSSA bacteremia in the original manuscript [16]. The reduction as demonstrated in the pooled estimate of O’Horo’s meta-analysis was caused by a decrease in BSI caused by coagulase-negative staphylococci only [30]. Two studies included in our review were excluded in O’Horo’s meta-analysis, one study because the outcome was colonization instead of infection [13]. The reason for exclusion of the second study investigating BSIs, albeit with MRSA only, is unknown [18]. Five studies, excluded for methodological reasons in our study were included in O’Horo’s study. In 4 of these (from a total of 12 studies) statistically significant effects were obtained. Though the authors touch upon the subject of heterogeneity in their discussion, they do not comment on their reasons for pooling data. In summary, both the present study and O’Horo’s meta-analysis suggest an effect of CHG-BW on BSIs. Our study adds that the benefit for preventing BSI is limited to gram-positives (VRE and possibly MRSA) and that evidence for gram-negatives is lacking. Moreover, our findings also suggest that colonization with gram-positives is reduced by CHG-BW.

## Conclusions

Based on this systematic review we conclude that there is evidence that CHG-BW is effective in preventing carriage, and possibly BSI, with MRSA and VRE in ICU patients, although this evidence is weakened by inter-study differences in intervention, co-interventions, and patient case mix. Overall, the quality of the studies was good, with low to medium risk of bias. There was no evidence (or lack of evidence) that CHG-BW reduces acquisition of carriage or infections with ARGNB. Future studies should address the effects of CHG-BW on acquisition of carriage and infections with ARGNB, preferably by investigating the effects of CHG-BW with the ICU as level of inference to account for colonization pressure, for instance by applying an ITS design with sufficient data points or a cluster-randomized trial design.

## Appendix: Research protocol

*Objective:* To evaluate the evidence for the effectiveness of the use of chlorhexidine body washings in reducing colonization and infection with AMRB in adult ICU patients.

*Databases to be searched:* PubMed, Embase, CINAHL, and OpenSigle from their inception until 1 April 2011. The search was last performed on 15 September 2011.

*Population:* Adult ICU patients.

*Intervention:* Chlorhexidine body washings.

*Outcomes:* All outcomes related to colonization, infection, and/or bacteremia with AMRB.

*Study design:* All.

*Free text search terms:* chlorhexidine, chlorhexidine gluconate, critical care, icu, intensive care, critical* ill, critical* illness, intensive treatment unit*, hospital, hospitals, inpatient*, hospitalis*, hospitaliz*.

*MeSH terms:* Chlorhexidine (“Chlorhexidine”[Mesh]), Chlorhexidine gluconate (“Chlorhexidine gluconate”[Substance Name]).

*Study selection (after removing duplicates):* Screen title and abstract of all identified articles for relevance, without blinding to journal and authors, by two independent reviewers (LD and MD). In case of discordant results consensus by discussion with a third reviewer (MB).

(A) Inclusion

Body washing with CHG as an intervention to control AMRB.

Colonization or infection with MRSA or VRE or ARGNB or any combination of those microorganisms or clearance of colonization with these micro-organisms as an outcome.

Setting: ICU or hospital without explicit absence of an ICU.

Patient population: Adults.

Non-English language papers and non-published papers all accepted.

Reviews included.

Letters to scientific journals included.

(B) Exclusion

Outbreak reports (defined as an increase in incidence that lasted <6 months) excluded as having weak evidence.

Chart reviews excluded.

Studies specifically on the subject of oral or topical decontamination, and studies on hand hygiene only excluded.

*PubMed search strategy:* [(critical care unit* OR ccu NOT coronary care unit*) OR critical care OR cc OR intensive care unit* OR icu OR intensive care OR ic OR (critical* AND ill) OR (critical* AND illness) OR intensive treatment* OR (intensive treatment* AND unit)* OR itu OR hospital OR hospitals OR inpatient* OR hospitaliz* OR hospitalis*] AND (chlorhexidine gluconate* OR chlorhexidine* OR body wash*).
